# Late Airway Compromise Secondary to Dural Tear and Cerebrospinal Fluid Leak

**DOI:** 10.31486/toj.23.0026

**Published:** 2023

**Authors:** Larry R. Hutson, Russell K. McAllister

**Affiliations:** Department of Anesthesiology, Baylor Scott & White Medical Center–Temple, Temple, TX

**Keywords:** *Airway management*, *airway obstruction*, *cerebrospinal fluid leak*, *cervical vertebrae*, *neck*

## Abstract

**Background:** While dysphagia after anterior cervical spine surgery is common, a dural tear is a rare complication. Airway compromise resulting from cerebrospinal fluid collection is an even rarer complication that has only been described to occur in the first few days postoperatively.

**Case Report:** A 55-year-old male presented with progressive dysphagia and respiratory compromise 3 weeks after anterior cervical discectomy and fusion surgery at C3-C6. Imaging demonstrated extensive fluid collection in the retropharyngeal space and lateral neck, resulting in displacement of the cricoid cartilage rightward and anteriorly while also narrowing the pharyngeal space. After the patient's airway was secured by awake fiberoptic intubation, the fluid was determined to be cerebrospinal fluid (CSF) from a cervical dural tear. The tear was identified and repaired. The patient was extubated the next day, and a lumbar drain was placed to reduce the strain on the repair. After 11 days in the hospital, the patient made a full recovery.

**Conclusion:** Dural tears following cervical disc surgery are rare and almost always identified in the immediate postoperative period; however, a dural tear should still be considered when a patient presents with a fluid collection at a later date. While techniques for securing the airway would not be different based on the type of fluid, knowing that the fluid collection is CSF could prompt the anesthesia team to place a lumbar drain.

## INTRODUCTION

Anterior cervical spine surgery can be associated with a host of potential complications, and although dysphagia after surgery is common, a cerebrospinal fluid (CSF) leak from a dural tear is not.^1^ Airway compromise is even rarer in patients following cervical spine surgery, especially compromise requiring emergent intervention to prevent complete airway obstruction.^2^ While this complication has previously been described during the early postoperative phase (1 to 4 days),^3^ to our knowledge, we present the first report of delayed presentation of airway compromise caused by a CSF leak from a dural tear following anterior cervical discectomy and fusion (ACDF). The CARE checklist was used to facilitate the writing of this manuscript. Written Health Insurance Portability and Accountability Act authorization from the patient was obtained for this publication.

## CASE REPORT

A 55-year-old male with a medical history significant for hypertension, diabetes mellitus type 2, tobacco abuse, and morbid obesity (body mass index of 44 kg/m^2^) presented for elective ACDF. He had a 3-year history of symptomatic deltoid and bicep weakness accompanied by paresthesias in the form of numbness in his hands. Imaging determined the symptoms were secondary to progressive spinal cord compression with bilateral foraminal stenosis that was most pronounced at the C3-C4 level. His symptoms had worsened significantly following a motor vehicle accident 3 weeks prior to his surgery.

The patient underwent a successful C3-C6 ACDF and was extubated at the conclusion of surgery. On postoperative day 1, the patient reported dysphagia that resolved spontaneously during the subsequent 48 hours. On postoperative day 3, he was discharged home with marked improvement in his initial upper extremity symptoms. He was also given a referral for an outpatient sleep evaluation because of concerns for undiagnosed obstructive sleep apnea.

Three weeks postsurgery, prior to a scheduled 1-month follow-up appointment, the patient presented to the emergency department because of a 2-week history of a progressively enlarging bulge in his anterior neck that resulted in a return of his dysphagia, beginning with solids and progressing to liquids. On physical examination, he remained in a cervical collar and had no obvious neurologic deficits. However, swelling under the cervical collar with rightward displacement of the cricoid cartilage and hyoid was noted, and the patient exhibited orthopnea.

**Figure 1.**
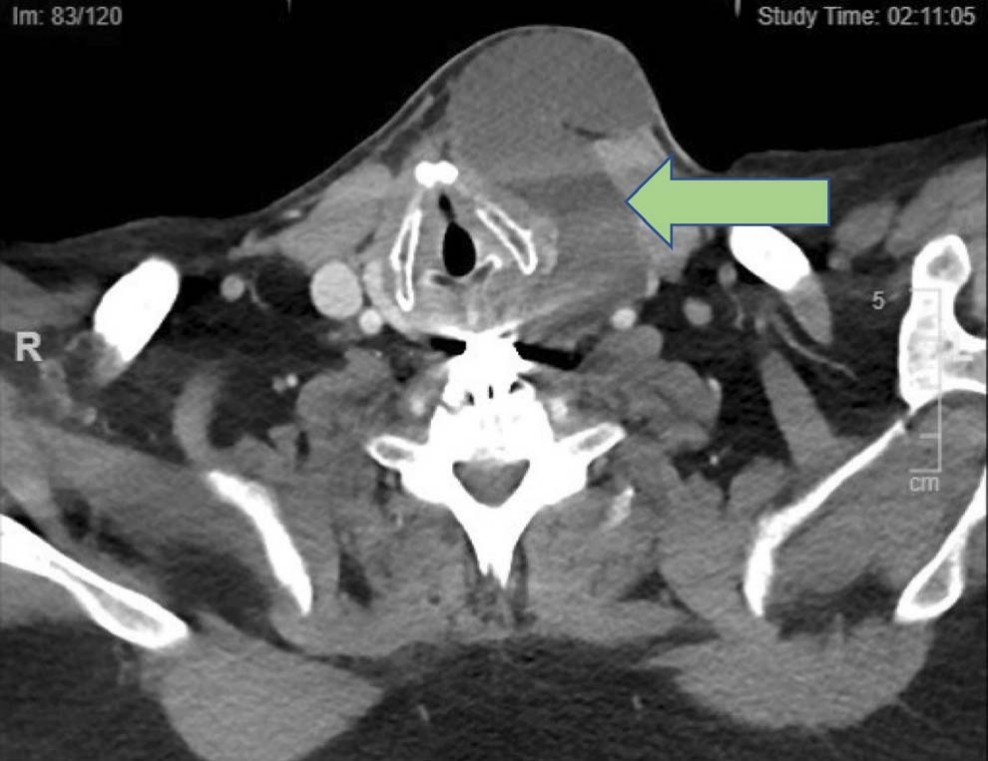
Axial computed tomography image of the neck. Green arrow indicates large cerebrospinal fluid collection emerging from the retropharyngeal space extending to the left of the cricoid cartilage and displacing it rightward, with resulting large anterior mass effect and superior tracheal compromise.

**Figure 2.**
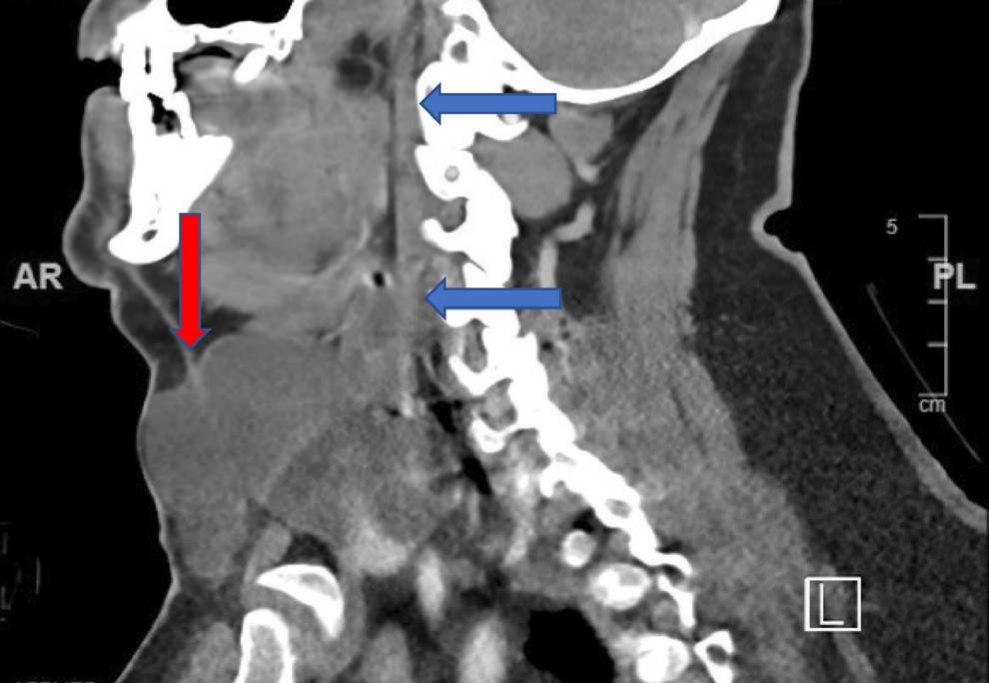
Sagittal computed tomography image of the neck. Blue arrows indicate extensive craniocaudal retropharyngeal cerebrospinal fluid (CSF) collection. Red arrow indicates large CSF collection encompassing the cricoid cartilage with anterior mass effect and tracheal compromise.

Computed tomography scan of the head and neck demonstrated fluid collection in the retropharyngeal space, anterior to the recent ACDF. The radiologist reported that the fluid collection was consistent with postoperative hematoma or seroma. The collection measured 6.3 cm craniocaudally, 4.5 cm transversely, and 1.5 cm in the anteroposterior direction, resulting in a significantly narrowed pharyngeal space. The fluid collection also spread to the left side of the patient's neck, creating a mass effect on the trachea that displaced it rightward and anteriorly (Figures 1 and 2).

Because of the patient's orthopnea, he was unable to tolerate the supine position, so he was urgently transported to the operating room in the 90-degree upright position. His oropharynx was anesthetized with 4% lidocaine through an atomizer. Midazolam 0.5 mg and glycopyrrolate 0.2 mg were administered intravenously. A nasal trumpet fitted with a 7.0 endotracheal tube adapter was coated with lidocaine ointment and inserted into a naris. The anesthesia circuit was attached to the nasal trumpet and adapter, and oxygen 10 L/min with 0.5% sevoflurane was administered.

Video laryngoscopy D-blade (Karl Storz SE & Co KG) was used, but vocal cords were not initially visualized because of the anterior and rightward shift of the structures from the mass effect. A flexible bronchoscope loaded with an 8.0 endotracheal tube was inserted alongside the video laryngoscope, and the 2 devices were used concurrently to guide the flexible bronchoscope into the trachea on the first pass. The endotracheal tube was passed into the trachea, and general anesthesia was induced with intravenous propofol.

Surgical exploration identified the fluid collection as CSF. A cervical dural tear was visualized and repaired. The defect was of sufficient size to require a dural patch. The surgeon then proceeded with a revision of the ACDF that resulted in a C5-C6 partial corpectomy.

At the completion of surgery, the patient remained intubated and was transported to the intensive care unit for further monitoring. The following morning, the patient demonstrated a significant reduction in neck swelling and met extubation criteria. He was successfully extubated, and a lumbar drain was placed to reduce the pressure on the newly patched dura. The remainder of his hospital course was uneventful, and his lumbar drain was removed on postoperative day 9. He remained in the hospital for 2 more days to ensure he had no other complications before discharge home. At follow-up in clinic 1 week after leaving the hospital, the patient had no residual dyspnea, dysphagia, or neurologic deficits.

## DISCUSSION

Surgery of the anterior cervical spine necessitates retraction of the trachea and the esophagus to the contralateral side to allow exposure of the surgical site. While difficulties with swallowing (>60% of patients) and hoarseness (50% of patients) are common after anterior cervical spine surgeries, airway complications are far less common.^1^ However, when airway compromise does occur, it typically is seen in the first 24 to 36 hours and may be severe enough to require emergent intubation or even emergent tracheostomy.^1^

Only a few studies examine airway compromise following anterior cervical spine surgery.^1-4^ Emery et al found airway compromise in 7 of 135 patients who underwent such a surgery (5.2% incidence).^4^ A larger retrospective study of 311 patients after anterior cervical spine surgery found an overall airway complication rate of 6.1% (19/311), although only 6 patients required reintubation (1.9%).^1^ Another retrospective review of 774 postsurgical patients demonstrated a similar reintubation rate of 1.8% (14/774).^3^

In the retrospective review by Sagi et al, the most common cause of airway compromise was acute pharyngeal edema, followed by hematoma.^1^ Relevant risk factors for postoperative reintubation were longer operative times (exceeding 5 hours) and exposure of 4 or more vertebral bodies. Additionally, exposure of C4 or above was also predictive of postoperative airway compromise, with the prevailing theory being that approaching the mandible requires a more forceful retraction, thus leading to a greater amount of pharyngeal trauma and resultant edema.^1^ The retrospective study by Li et al found a statistically significant correlation between airway compromise and surgical involvement of higher cervical segments.^3^ Surgery at C5 or above was associated with a 2.5 times higher incidence of postoperative airway complications compared to patients who underwent anterior cervical surgery at C6 or below**.** In a prospective study of 87 anterior cervical spine surgery patients examined by radiograph preoperatively, immediately postoperatively, and again 5 days after surgery, Suk et al found that patients who had operative sites in the C2-C4 region showed significant degrees of tissue swelling, even if no airway complications were noted.^5^

A dural tear following anterior cervical spine surgery is a rare occurrence (0.6% incidence).^6^ Most dural tears are identified during the initial surgery and repaired at that time. In a large, multinational study of cervical dural tears, most patients required no further treatment after the initial repair. However, 12% required additional intervention, with the majority of those undergoing an operative revision dural repair. Most patients were prescribed bed rest for several days, and a small number (14%) had a lumbar drain in place for several days.^6^

Our review of the literature identified 2 reports (3 patients total) of a dural tear resulting in a CSF accumulation severe enough to result in airway compromise.^7,8^ In the case series by Chang et al, 1 patient presented with respiratory problems on postoperative day 1 and the other patient presented on postoperative day 4.^7^ In the case reported by Penberthy and Roberts, the patient presented with respiratory distress and stridor 48 hours postoperatively.^8^ All 3 patients underwent successful surgical repair of the CSF leak and had no further issues. Our patient had an increased amount of swelling that began approximately 1 week postoperatively but was masked to some degree by the cervical collar. However, his initial symptoms of airway compromise did not present until 3 weeks postoperatively, prompting him to seek care at the emergency department.

## CONCLUSION

While cervical spine surgery–related airway compromise attributable to a CSF collection from a dural tear has been described in rare circumstances, presentation with respiratory distress in the relatively late timeframe of 3 weeks postoperatively has not been previously described to our knowledge. The medical team must have an understanding of the rare but real possibility of late airway compromise caused by a CSF leak and accumulation that can require emergent intervention.

## Notes

*This article meets the Accreditation Council for Graduate Medical Education and the American Board of Medical Specialties Maintenance of Certification competencies for Patient Care and Medical Knowledge.*
